# Coping with Covid-19: stress, control and coping among pregnant women in Ireland during the Covid-19 pandemic

**DOI:** 10.1186/s12884-022-04579-1

**Published:** 2022-04-01

**Authors:** Sarah Crowe, Kiran Sarma

**Affiliations:** National University of Ireland, Galway, Ireland

**Keywords:** *Pregnancy*, *Covid-19*, *Coping*, *Control*

## Abstract

**Background:**

The aim of the current study is to investigate the relationship between perceived control, coping and psychological distress among pregnant women in Ireland during the Covid-19 pandemic. It is hypothesised that lower levels of perceived control, greater use of avoidant coping and greater Covid-19 related pregnancy concern will be associated with psychological distress. In addition, it is hypothesised that the relationship between Covid-19 related pregnancy concern and psychological distress will be moderated by perceived control and avoidant coping.

**Method:**

The study is cross-sectional, utilizing an online questionnaire, which was completed by 761 women in January 2021. The questionnaire includes measures of perceived control, coping style, perceived stress, anxiety and depression.

**Results:**

Correlation analyses found that lower levels of perceived control were associated with higher levels of avoidant coping and psychological distress. There was also a significant positive relationship between avoidant coping and psychological distress. Using multiple regression, perceived control, avoidant coping and Covid-19 related pregnancy concern were found to predict 51% of the variance in psychological distress. However, in the moderation analysis, perceived control and avoidant coping were not found to moderate the relationship between Covid-19 related pregnancy concern and psychological distress.

**Conclusion:**

The results from this study suggest that pregnant women in Ireland are experiencing increased levels of psychological distress during the Covid-19 pandemic. The findings also suggest that perceptions of control and avoidant coping are associated with psychological distress in this group and could be used as intervention targets.

## Background

For many women pregnancy is experienced as a time of joy and excitement. However, pregnant women can also experience significant stress. Stress can be defined as any demand in the environment which exceeds the individuals adaptive capacity, resulting in physical or psychological strain [1]. A wealth of literature exists on the deleterious effects of emotional distress, life event stress and disaster exposure experienced in the prenatal period. Stress in the form of adverse life events has consistently been identified as one of many predictors of post-partum depression [2, 3]. Higher levels of perceived stress have also been identified as a predictor of depression and anxiety in the prenatal period [4, 5]. For the developing infant, high levels of prenatal stress have been linked to impaired fetal growth and pre-term delivery [6–8], as well as negative outcomes for cognitive, emotional and physical development [9, 10].

The Covid-19 pandemic presented a unique and unprecedented stressor in the lives of pregnant women. In addition to common stresses associated with Covid-19 such as reduced social contact, worry about becoming infected, and financial concerns [11], pregnant women faced altered and often reduced prenatal care [12, 13]. They worried about how a potential Covid-19 infection could adversely impact their pregnancy [13, 14]. They also reported feeling unprepared for the birth [13] and were concerned about not having a partner present during labour due to Covid-19 restrictions [15]. A number of studies have demonstrated that pregnant women experienced increased levels of psychological distress than prior to the pandemic [16–18]. One study found that 26% of pregnant women in Ireland displayed clinically significant symptoms of depression during June and July of 2020 [19]. This is compared to the average prevalence rate for prenatal depression which is believed to be between 10–15% [20] and was found to be 16% in Ireland prior to the pandemic [20].

The Transactional Model of Stress and Coping [21] is a cognitive model which describes stress as an interaction between the person and their environment. The model describes a process in which an environmental stressor triggers two stages of cognitive appraisal followed by the selection of a coping response, and forms the basis of much contemporary stress and coping research [22].

In the Transactional Model, coping is defined as any effort, for example cognitive or behavioural, employed to reduce the threat introduced by a stressor [21]. Coping has been identified as a mediating factor in the relationship between stress and distress, whereby successful coping is associated with less physical and psychological strain [21]. Folkman and Lazarus categorised coping strategies into problem-focused or emotion-focused, based on their function. In general, problem-focused coping refers to efforts to control the environment, directing attention to the stressor itself. Whereas emotion-focused coping refers to attempts to modify the emotional impact of the stressor, for example by using humour or turning to religion. Other researchers have proposed alternative categorisations of coping, which typically include a category related to dealing with the stressor directly, a category related to managing one’s emotions and an additional category which is less adaptive [23–25]. These less adaptive responses often include avoiding the stressor and its emotional impact and are frequently associated with greater psychological distress [26–28].

Perceived control is an example of a cognitive appraisal in Lazarus and Folkman’s model and therefore effects the coping response selected [29]. Individuals high on perceived control are more likely to believe that they have the ability to produce desired outcomes and avoid undesired ones through their own actions. Researchers have suggested that higher perceived control leads to the use of more problem-focused coping, while lower perceived control leads to the use of more emotion-focused coping [29–32]. Higher perceived control has also been associated with less avoidant coping [27]. Perceptions of control can mediate the relationship between stress and psychological distress [33, 34], whereby having a greater sense of perceived control is associated with lower distress and better outcomes [35–37].

Research on coping during pregnancy has found that avoidant coping styles are more frequently associated with psychological distress, while problem-focused and emotion-focused coping are more often associated with well-being [38, 39]. In a study of pregnant women who experienced stress in relation to being from an ethnic minority and low-income background, Rudnicki et al. [40] found that avoidant coping was correlated with depressed mood during pregnancy. In a longitudinal study of primiparous women, Honey et al. [41] found that higher levels of avoidant coping during the last trimester predicted higher rates of depression at three weeks postpartum, even when a history of depression was controlled for. In their regression analysis, which included the additional predictors of stress, perceived control and social support, 49% of the variance in depression scores was explained. There are also findings which suggest that avoidant coping during pregnancy can have a negative impact on birth outcomes [42, 43]. However, these findings have not been widely replicated.

While problem-focused and emotion-focused coping are more often associated with well-being [38], the findings are somewhat inconsistent. Some studies have suggested that emotion-focused coping is associated with greater distress during pregnancy [44, 45], while others have suggested that problem-focused coping is [46, 47].

Higher levels of perceived control have also been associated with greater well-being and less psychological distress among pregnant women [48–50]. In a study looking at infertility and IVF, Gourounti et al. [51] found that perceptions of controllability were negatively associated with avoidant coping. Furthermore, they found that having a low sense of personal control in combination with the use of avoidant coping was associated with increased stress and anxiety. In their regression analysis, age, perceived control and coping style predicted 38% of the variance in depressive symptomology.

### The current study

The aim of the current study is to investigate the relationship between perceived control, coping and psychological distress among pregnant women in Ireland during the Covid-19 pandemic.

Throughout the pandemic in Ireland, alterations have been made to prenatal care. Face-to-face appointments have been reduced in favour of virtual consultations, with antenatal classes also taking place online [52, 53]. While regulations vary across hospital groups, some sites have imposed restrictions on having partners present for prenatal scans and during labour [52]. Restrictions on social contacts also mean that women can not celebrate and share their pregnancy experience with friends and family, or socialise in-person with other pregnant women for support [53].

A number of studies have been published indicating that pregnant women are experiencing increased psychological distress than prior to the pandemic [16–19]. Research has also focused on how various forms of coping impact mental wellbeing [54–58]. However, to the author’s knowledge, no specific studies have been published which focus on how perceived control and coping are associated with psychological distress among this population, particularly in an Irish context. A greater understanding of this relationship will help to identify women who may be particularly at risk and to inform interventions such as stress management and coping skills training.

It is hypothesised that lower levels of perceived control, greater use of avoidant coping and greater Covid-19 related pregnancy concern will be associated with psychological distress. In addition, it is hypothesised that the relationship between Covid-19 related pregnancy concern and psychological distress will be moderated by perceived control and avoidant coping.

## Method

### Design

The study was cross-sectional, utilizing an online questionnaire.

### Participants

The online questionnaire was completed by 985 participants. Data analyses were conducted using only complete responses, leaving a final sample of 761 participants. Chi Squared tests indicated that non-completers differed significantly from completers with regard to employment status χ2 (6, *n* = 965) = 17.44, *p* = 0.008, Cramer’s V = 0.134, and trimester χ2 (2, *n* = 958) = 10.49, *p* = 0.005, Cramer’s V = 0.105. The two groups did not differ significantly with regard to any of the other demographic factors under investigation.

All participants were pregnant women, 18 years of age or older, who resided in the Republic of Ireland. Demographic characteristics of the sample are presented in Table 1. It is notable that 7% of the sample had contracted Covid-19, as this is comparable with the overall rate of approximately 5% in the total population of the Republic of Ireland at the time [59].

**Table 1:** Characteristics of Study Population

	*N* = 761 n (%)
	(*N* = 761)
First Pregnancy	
Yes	279 (36.7)
No	482 (63.3)
Trimester	
First	79 (10.4)
Second	311 (40.9)
Third	366 (48.1)
Ethnic Background	
Irish	728 (95.7)
Irish Traveller	0
Other Caucasian	25 (3.3)
Other Asian Background	9 (.1)
Other inc. Mixed Background	7 (.9)
Relationship Status	
In a Relationship	65 (8.5)
Cohabiting	121 (15.9)
Married	553 (72.7)
Single	7 (.9)
Employment Status	
Unemployed	18 (2.4)
Homemaker/Carer	60 (7.9)
Unable to Work	8 (1.1)
Student	4 (.5)
Self-Employed	36 (4.7)
Employed Full Time	568 (74.6)
Employed Part Time	67 (8.8)
High Risk Pregnancy	
Yes	206 (27.1)
No	405 (53.2)
Ever Contracted Covid – Self	
Yes	53 (7)
No	708 (93)
Ever Contracted Covid – Other in Household	
Yes	47 (6.2)
No	741 (93.8)

### Measures

#### Demographics

Participants were asked about demographic information including age, ethnicity, relationship status and employment status. They were also asked about their experience of the pandemic, for example if they or someone in their household had contracted Covid-19. If it was not their first pregnancy participants were asked about their previous pregnancy and delivery experience(s).

#### Coping

The Brief COPE [60] is a measure of dispositional coping style, with 28 items which give rise to 14 sub-scales. The scale uses a 4 point Likert from “I usually don’t do this at all” to “I usually do this a lot”. Items include “I get emotional support from others” and “I give up trying to deal with it”. The scale has strong internal reliability [60], and has also been found to have acceptable reliability in pregnant samples [38]. In the current study the Cronbach’s alpha for the total scale was 0.78. For this study, the 14 sub-scales were grouped into three categories: problem-focused (active coping, planning, using instrumental support), emotion-focused (positive reframing, acceptance, humour, religion, use of emotional support) and avoidant coping (self-distraction, denial, venting, substance use, behavioural disengagement, self-blame). This categorisation has been commonly used including in pregnancy research [61], and in the current study the subscales showed good internal consistency with alphas of 0.70, 0.78 and 0.62 respectively.

#### Sense of control

The MIDI Sense of Control [62] is a 12 item scale which measures perceived sense of control. It is a 7 point Likert scale with answers ranging from “strongly agree” to “strongly disagree”. Items include “I often feel helpless in dealing with the problems of life” and “what happens to me in the future mostly depends on me”. Scores range from 12–84 with higher scores indicating greater levels of perceived control. It has strong reliability [62], and the Cronbach’s alpha for current study was 0.83.

#### Anxiety

The Generalised Anxiety Disorder-7 (GAD-7) was used to measure anxiety [63]. This is a 7 item scale which asks participants to rate their anxiety over the past 2 weeks on a 4 point Likert scale with answers ranging from “not at all” to “nearly every day”. Items include “worrying too much about different things” and “trouble relaxing”. Total scores range from 0 to 21 (0–4 = minimal anxiety, 5–9 = mild anxiety, 10–14 = moderate anxiety, 15–21 = severe anxiety). The scale has been found to be valid and reliable for both clinical and research purposes [63]. In the current study the Cronbach’s alpha was 0.91.

#### Depression

The Edinburgh Postnatal Depression Scale (EPDS) [64] was used to measure depression. This is a 10 item screening tool which is commonly used in the prenatal period and up to one year postpartum. The scale asks about how respondents have felt over the past seven days and items include “I have felt sad or miserable” and “I’ve been so unhappy that I’ve been crying”. There are 4 possible responses for each item with corresponding scores ranging from 0–3. Total scores range from 0–30, with a score of 13 or above indicating clinically significant symptoms of depression. The EPDS has been found to be reliable and valid in pregnant samples [65]. The Cronbach’s alpha for current study was 0.87.

#### Perceived stress

Perceived stress was measured using the Perceived Stress Scale [66]. This is a 10 item scale asking about stress over the past month, using a 5 point Likert from “never” to “very often”. Items include “In the last month, how often have you felt difficulties were piling up so high that you could not overcome them?”. Total scores range from 0 to 40 (0–13 = low stress, 14–26 = moderate stress, 27–40 = high stress). Cohen et al. [66] found the scale to have a Cronbach’s alpha of 0.78 and it has also been shown to have good reliability in pregnant samples [67]. In the current study the Cronbach’s alpha was 0.88.

#### Prenatal stress

The Prenatal Distress Questionnaire [47] is a 12 item scale assessing aspects of pregnancy which some women may find uncomfortable or upsetting. The PDQ has been found to be a valid and reliable measure of stress relating to pregnancy [67]. In the current study the Cronbach’s alpha was 0.84. This scale was used to assess for convergent validity with the Covid-19 related Pregnancy Concern scale which was developed for the current study and was not used in the main study analyses.

#### Psychological distress

As total scores on the EPDS, GAD-7 and PSS were highly correlated (rs > 0.7, ps < 0.001) the three measures were standardised and then combined in order to create a latent variable of total Psychological Distress. This total Psychological Distress scale had a Cronbach’s Alpha of 0.95.

#### Covid-19 related pregnancy concern

An 8-item questionnaire was developed for the current study in order to investigate participants concerns in relation to being pregnant during the pandemic. The items for this scale were determined by reviewing research articles which explored pregnant women’s main concerns about the pandemic [14, 53, 68]. Participants were asked about worries such as contracting the virus and transmitting it to the baby, and how the pandemic may affect their pregnancy and delivery experience. Items were scored on a 5 point Likert scale from “Not at all worried” to “Extremely worried”. Total scores range from 8–40, with higher scores indicating greater levels of concern. The Cronbach’s alpha of the scale was good at 0.89. The scale also showed convergent validity with the Prenatal Distress Questionnaire (*r* = 0.40) and the Generalised Anxiety Disorders-7 (*r* = 0.47).

A principal axis factoring analysis was conducted on the 8 items of the Covid-19 Related Pregnancy Concern scale with oblimin rotation (promax). Inspection of the correlation matrix revealed that all of the coefficients were above 0.3. Bartlett’s Test of Sphericity X^2^ (761) = 3237.99, *p* < 0.001, supported the factorability of the correlation matrix. The Kaiser–Meyer–Olkin value was 0.88 verifying sampling adequacy [69].

Principal axis analysis revealed the presence of two factors with eigenvalues exceeding 1, explaining 51.84% and 10.05% of the variance respectively. Inspection of the point of inflexion on the scree plot also justified the retention of two factors. This was further supported by the results of a Parallel Analysis, which showed only two components with eigenvalues exceeding the corresponding criterion values for a randomly generated data matrix of the same size (8 variables × 761 respondents).

Table 2 shows the factor loadings after rotation. The items that cluster on the same factors suggest that factor 1 represents concern about the effects of Covid-19 on the pregnancy experience, and factor 2 represents concern about becoming infected with Covid-19.

**Table 2:** Pattern and Structure Matrix for Principal Axis Analysis Covid-19 Related Pregnancy Concern Items

Item	Pattern Coefficients		Structure Coefficients	
	Factor 1—Effect on Pregnancy Concern	Factor 2—Infection Concern	Factor 1—Effect on Pregnancy Concern	Factor 2—Infection Concern
1. Worry about contracting Covid-19 myself	-.055	**.878**	.501	**.843**
2. Worry about a loved one contracting Covid-19	-.048	**.802**	.461	**.772**
3. Worry about transmitting Covid-19 to my baby	.049	**.747**	.522	**.778**
4. Worry that Covid-19 will have a negative effect on my pregnancy experience	**.414**	.389	**.661**	.652
5. Worry that Covid-19 will have a negative effect on my delivery experience	**.606**	.206	**.737**	.590
6. Worry that Covid-19 will have a negative effect on my postpartum experience	**.906**	-.074	**.859**	.500
7. Worry that Covid-19 will have a negative impact on my ability to access social support during and after my pregnancy	**.910**	-.142	**.820**	.434
8. Worry about my baby living in the world with Covid-19	**.543**	.236	**.692**	.580

***Note:*** Major loadings for each item appear in bold

These factors are similar to those identified by Preis et al., [70] in their factor analysis of the Pandemic-Related Pregnancy Stress Scale (PREPS) in that one factor is related to concerns about becoming infected with Covid-19 (PREPS Infection) and another factor is related to concerns about how Covid-19 and the resulting restrictions may affect the prenatal and postnatal experience (PREPS Preparedness). The PREPS also includes a third factor (PREPS Positive Appraisal) which looks at the positive elements of being pregnant during the pandemic, for example being pregnant giving mothers strength to get through the hardship of the pandemic.

### Procedure

Cross-sectional data were collected from the 10^th^ to the 31^st^ of January 2021. This was during a significant peak of Covid-19 infection in Ireland when a nationwide lockdown was in effect. Potential participants were targeted through social media, in particular Irish based accounts and sites which were aimed at the target demographic of pregnant women. The questionnaire was completed using the online survey host “Qualtrics”. Participants read an information sheet and provided consent, before filling in the questionnaire which took approximately 10–20 min to complete. Participants were informed of their right to withdraw from the study at any time.

### Statistical analysis

Data analysis was completed using Statistical Package for Social Sciences (SPSS) version 26 [71]. Prior to hypothesis testing data screening and descriptive analyses were carried out. Data were also assessed for normality, outliers and homoscedasticity prior to analysis. The psychometric properties of the scales were assessed using Cronbach’s Alpha. A series of t-tests and analysis of variance were used to investigate the relationship between demographic factors and psychological distress. The first study hypothesis was investigated using a series of hierarchical regressions. The second hypothesis was investigated using a moderation model utilizing Hayes’s PROCESS macro add-on for SPSS version 26 [72].

## Results

### Descriptive statistics

A summary of the descriptive analysis across the variables which make up the distress composite can be seen in Table 3. The mean score on the EPDS was 12.15 which is just below the cut-off score of 13 that indicates clinical significance. However, 43% of the sample did score in the clinically significant range. On the GAD-7, the mean score was in the mild anxiety range (scores of 5–9) and 20% of the sample scored in the moderate to severe range (scores of 10–21). On the PSS the mean score was in the moderate range (scores of 14–26), and 7.5% of participants scored in the high stress range (scores of 27–40).

**Table 3:** Descriptive Statistics of Psychological Variables

Measure	Mean	SD
Perceived Stress (PSS)	18.14	5.88
Anxiety (GAD-7)	7.53	5.08
Depression (EPDS)	12.15	5.14

### T-Tests and analysis of variance

A series of t-tests and analyses of variance were carried out to explore differences across groups in relation to demographic factors and psychological distress. The demographic factors of interest were first pregnancy, trimester, relationship status, high risk pregnancy, previous pregnancy experience, previous delivery experience, and a history of contracting Covid-19 oneself or within the household.

There was a statistically significant difference in distress scores between women who had a high risk pregnancy (M = 39.51, SD = 15.47) and those who did not (M = 36.46, SD = 14.76), t(609) = -2.37, *p* = 0.018. The effect size was small with an eta squared value of 0.009 (0.01 = small effect, 0.06 = moderate effect, 0.14 = large effect) [73]. There was also a statistically significant difference in distress scores between groups by relationship status, F (3, 742) = 3.58, *p* = 0.01. Post-hoc analysis using Tukey HSD test found that the mean for women who were married (M = 36.79, SD = 14.83) was significantly different from the mean for women who were in a relationship (M = 41.77, SD = 15.17). The effect size was small with an eta squared value of 0.01.

Among women who had previously been pregnant (*n* = 486), there was a statistically significant inter-group difference regarding how difficult they found the previous pregnancy F (3, 482) = 7.42, *p* < 0.001. Post-hoc analysis indicated that mean distress scores differed significantly between women who found their past pregnancy/pregnancies very difficult (M = 43.20, SD = 15.69, *n* = 86) versus not at all difficult (M = 34.52, SD = 15.43, *n* = 153), and also between those who found them very difficult versus a little difficult (M = 38.32, SD = 13.04, *n* = 238). Once again the effect size was small with an eta squared of 0.04. Among women who had previously given birth (*n* = 446) there was a statistically significant inter-group difference regarding how difficult they found the birth(s) F (2, 443) = 12.02, *p* < 0.001. Post-hoc analysis revealed that mean distress scores differed significantly between those who found the birth(s) not at all traumatic (M = 34.84, SD = 14.93, *n* = 135) and very traumatic (M = 43.78, SD = 14.21, *n* = 96), and those who found the birth(s) a little traumatic (M = 36.98, SD = 13.44, *n* = 215) versus very traumatic. Once again the effect size was small with an eta squared value of 0.05.

With a large sample, small differences can more easily become statistically significant [74]. Therefore, it is important to look to effect sizes when determining the inclusion of demographic factors to be controlled for in the regression. It was suspected that due to the small effect sizes, these factors would not make a significant contribution to the regression models. However, regression analyses were initially carried out including the two factors with the largest effect size, namely previous pregnancy experience and previous delivery experience. These factors were found not to make a statistically significant contribution to the regression models and were therefore dropped from further analysis.

### Correlation analysis

Pearson correlations were conducted between the overall outcome variable of psychological distress and its component measures, and the participants scores on measures of perceived control, problem-focused, emotion-focused and avoidant coping. The results are presented in the correlation matrix below (Table 4).

**Table 4:** Results of Pearson Correlations Investigating Relationships Between Variables

	Mean	SD	1	2	3	4	5	6	7
1. Psychological Distress	.00	2.75	1						
2. Perceived Control	60.56	10.77	-.56^b^	1					
3. Problem-Focused Coping	15.96	3.43	-.20^b^	.33^b^	1				
4. Emotion-Focused Coping	23.35	4.19	-.20^b^	.23^b^	.61^b^	1			
5. Avoidant Coping	22.26	3.87	.48^b^	-.34^a^	.11^b^	.13^b^	1		
6. Covid-19 Infection Concern	11.28	2.66	.41^b^	-.22^b^	-.06	.06	.20^b^	1	
7. Covid-19 Pregnancy Concern	18.97	4.43	.49^b^	-.22^b^	-.05	-.10^a^	.29^b^	.61^b^	1

^a^ Correlation is significant at the 0.05 level (2-tailed)

^b^Correlation is significant at the 0.01 level (2-tailed)

As expected, total psychological distress was positively associated with avoidant coping, and the strength of this relationships was medium (*r* = 0.48, *p* < 0.001). Perceived control was negatively associated with psychological distress, and the strength of this relationship was large (*r* = -0.56, *p* < 0.001). Perceived control was positively associated with problem-focused (*r* = 0.33, *p* < 0.001) and emotion-focused coping (*r* = 0.23, *p* < 0.001) and was negatively associated with avoidant coping (*r* = -0.34, *p* < 0.001). These finding support the hypotheses that lower levels of perceived control and higher levels of avoidant coping are associated with greater levels of distress, and that lower levels of perceived control and higher levels of avoidant coping are associated with each other. Both problem-focused and emotion-focused coping had a small negative relationship with psychological distress (*r *= -0.20, *p* < 0.001; *r *= -0.20, *p* < 0.001), and had a large positive association with each other (*r* = 0.61, *p* < 0.001).

### Regression analysis

To investigate the first hypothesis, that lower levels of perceived control, greater use of avoidant coping and greater Covid-19 related pregnancy concern will be associated with psychological distress, a multiple linear regression was conducted (see Table 5). All of the predictor variables, namely perceived control, avoidant coping and Covid-19 related pregnancy concern, were found to be significantly associated with psychological distress in the correlation matrix and were therefor included in the regression analyses. As a two factor structure of the Covid-19 related pregnancy concern scale was supported through factor analysis, they were entered as separate variables in the regression analysis.

**Table 5:** Regression Analysis of Variables Predicting Psychological Distress

	Predictor Variable	B	SE	β	Sig	*R*^2^
Block 1						.51
	Constant	-2.23	.77		.000	
	Perceived Control Avoidant Coping	-.10 .18	.01 .02	-.39 .25	.000 .000	
	Covid-19 Infection Concern	.13	.03	.13	.000	
	Covid-19 Effect on Pregnancy Concern	.16	.02	.25	.000	

Multicollinearity was not detected as none of the IVs had a small tolerance value (< 0.10) or high VIF value (> 10) [75]. No major deviations from normality were detected as the points in the Normal P-P Plots followed a straight diagonal line. Therefore, the assumptions required for regression were met. A very small number of outliers could be observed in the Scatterplots (standard residuals of more than 3.3 or less than –3.3). However, due to the large sample size no action was taken [74].

In the regression analysis (Table 4), perceived control (β = -0.39, *p* < 0.001), avoidat coping (β = 0.25, *p* < 0.001), Covid-19 infection concern (β = 0.13, *p* < 0.001) and Covid-19 effect on pregnancy concern (β = 0.25, *p* < 0.001) significantly predicted perceived stress. The total variance explained by the model was 51%, F(4,756) = 198.2, *p* < 0.001.

### Moderation analysis

The second hypothesis, that the relationship between Covid-19 related pregnancy concern and psychological distress will be moderated by perceived control and avoidant coping, was investigated using moderation analysis with Hayes’s PROCESS macro in SPSS.

**Fig. 1:**
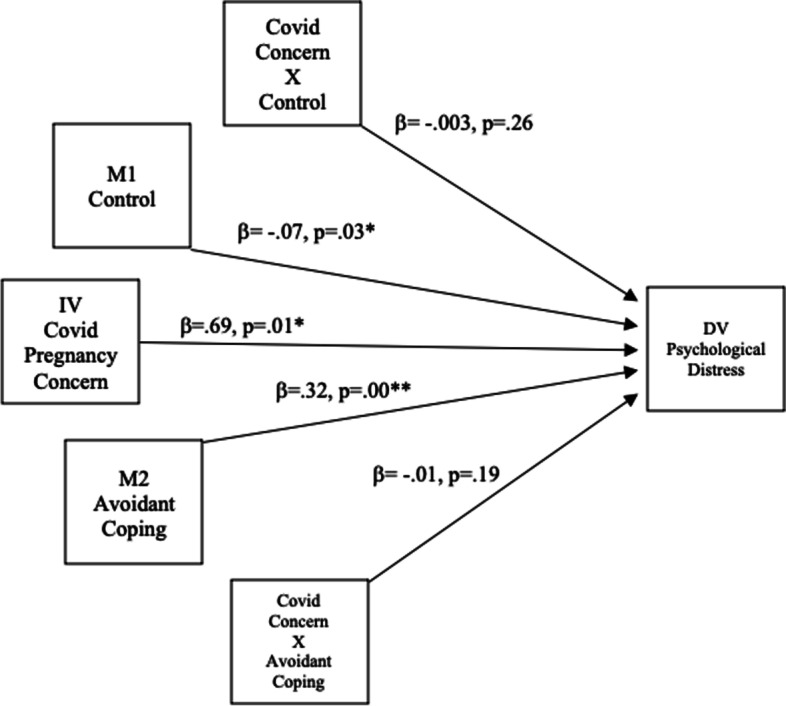
Moderating Effect of Control and Coping on Covid-19 Effect on Pregnancy Concern and Psychological Distress. * Significant at the 0.05 level. **Significant at the 0.01 level

**Fig. 2:**
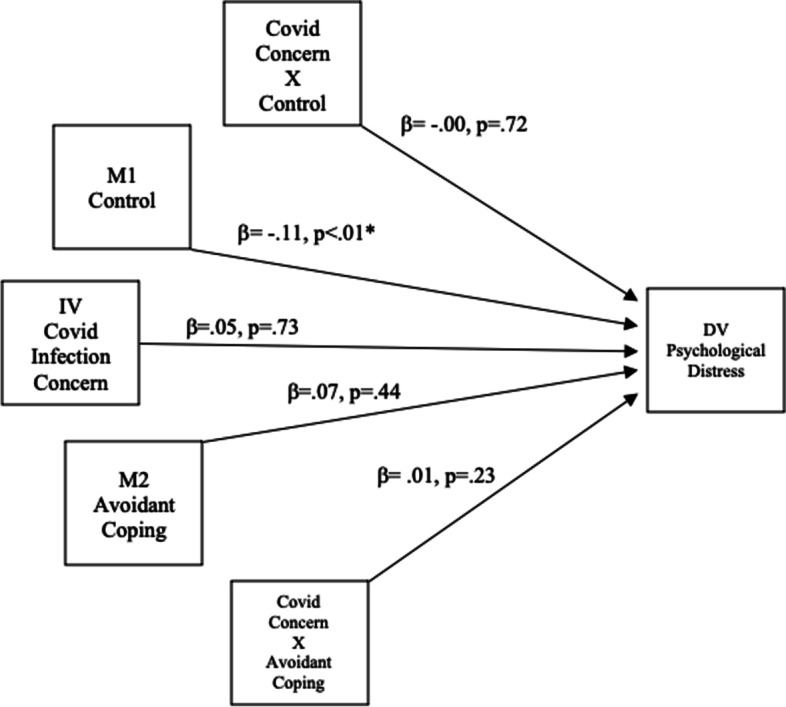
Moderating Effect of Control and Coping on Covid-19 Infection Concern and Psychological Distress. * Significant at the 0.05 level

Two separate analyses were carried out, using the two factors of the Covid-19 pregnancy concern scale, namely effect on pregnancy concern (Fig. 1) and infection concern (Fig. 2), as the independent variable. In both analyses perceived control was the first moderator, avoidant coping was the second moderator and psychological distress was the dependent variable. In both of these analyses, the relationship between Covid-19 related pregnancy concern and psychological distress was not found to be moderated by perceived control or avoidant coping. Therefore, the second hypothesis was not supported.

## Discussion

The aim of the current study was to investigate the relationship between perceived control, coping and psychological distress among pregnant women in Ireland during the Covid-19 pandemic. A number of recent studies indicated that pregnant women experienced increased psychological distress during the pandemic than prior to it [16, 17]. The current study sought to further knowledge in this area by exploring whether perceptions of control and coping style were associated with psychological distress among this population in an Irish context.

It was hypothesised that lower levels of perceived control would be associated with greater avoidant coping and increased psychological distress. This hypothesis was supported through correlation analyses which found significant negative relationships between perceived control and avoidant coping and perceived control and psychological distress, and a significant positive relationship between avoidant coping and psychological distress. Using multiple regression, perceived control, avoidant coping and the two factors of Covid-19 related pregnancy concern were found to predict 51% of the variance in psychological distress. However, the second hypothesis which stated that perceived control and avoidant coping would moderate the relationship between Covid-19 related pregnancy concern and psychological distress was not supported.

While this is the first study to look at control and coping among pregnant women in the context of Covid-19, other prenatal stress research has demonstrated similar findings. In a study of women undergoing IVF, Gourounti et al. found that low perceptions of controllability were associated with avoidant coping and with psychological distress. In their regression analysis, age, perceived control and coping style predicted 34% of the variance in fertility related distress, and 38% of the variance in depressive symptomology. Lobel et al. [76] also found that women with high-risk pregnancies who evaluated their situation as more controllable used less avoidant coping and were less distressed.

The findings also contribute to a recent body of research exploring how concerns about being pregnant during the Covid-19 pandemic have affected the mental well-being of pregnant women. Preis et al. [13] looked at pandemic-related pregnancy stress in a large sample of women in the U.S. and found that feeling unprepared for birth due to Covid-19 and fears of becoming infected with Covid-19 during pregnancy significantly predicted stress and anxiety. Similarly, Molgora and Accordini [15] found that expectations about how Covid-19 could negatively affect the childbirth experience were associated with increased anxiety in a sample of pregnant women in Italy. To the author’s knowledge, no other studies have been published looking at perceptions of control or coping in relation to concerns about pregnancy during the pandemic.

Both problem-focused and emotion-focused coping were negatively associated with all measures of psychological distress, and had a large positive association with each other. They were also both significantly and positively associated with perceived control. This finding supports previous research which suggest that that both problem and emotion-focused coping are associated with reduced levels of psychological distress during pregnancy [38, 39]. However, this finding does not support the suggestion that in situations perceived as less controllable emotion-focused coping is used more frequently [21], and is associated with less distress than problem-focused coping [61].

The findings from this study highlight the increased rates of psychological distress among pregnant women in Ireland during the pandemic. In this sample, 43% of participants scored above 13 on the EPDS, which is the cut-off for clinically significant symptoms of depression. This is higher than the rate of 26% observed in Ireland in another recent study [19] but is comparable to rates of 34% observed in Italy [15] and 41% in Canada [17]. These rates are much higher than the average prevalence rate for depression during pregnancy, which is believed to be between 10–15% [20, 77] and was found to be 16% in Ireland prior to the pandemic [78].

The findings from this study contribute to the wealth of research suggesting that pregnant women in Ireland and elsewhere are experiencing increased psychological distress than prior to the Covid-19 pandemic [15, 17, 19]. The findings also suggest that specific concerns about being pregnant during the pandemic are contributing to distress, as are lower levels of perceived control and the use of avoidant coping.

The importance of routine screening for women at risk of experiencing psychological distress during pregnancy in Ireland was highlighted by the National Maternity Strategy 2016–2026 [79]. Routine screening has been implemented in some maternity services, however, this procedure is still inconsistent [80]. As pregnant women appear to be experiencing increased distress during the pandemic, screening efforts should be heightened during this time. Simply being asked about their mental well-being during prenatal visits is frequently appreciated by pregnant women [81]. In keeping with Covid-19 restrictions, this could take place via mobile devices as this has been found to be a feasible approach for prenatal mental health screening [82].

There are a number of findings which suggest that perceived control and coping style are amenable to change through intervention [83–86]. Therefore, they could be useful intervention targets for pregnant women showing clinical levels of psychological distress during the pandemic and beyond. These interventions could use psychoeducation and CBT [83, 87, 88] as well as relaxation techniques like progressive muscle relaxation and deep breathing [89]. Again, these interventions could be delivered online or via mobile apps as these tools have been found to be useful among pregnant women [90–92] and in the context of Covid-19 [93].

To the author’s knowledge, this is the first study to investigate the relationship between perceived control and coping among pregnant women in the context of Covid-19. The novelty, along with the large sample size of 761, are strengths of the study.

The results from this study should be interpreted in light of a number of limitations. For example, the majority of participants in this study were married, with a third level education and there was very little ethnic diversity. Therefore, the results may not be generalisable to women from a low SES or ethnic minority background. It is also possible that there was a sampling bias as recruitment took place through online social media. Therefore, women who do not have access to the internet or have literacy difficulties may not be represented.

A future adjunct study should be carried out as approximately half of the current sample consented to being contacted for follow-up. This would allow for longitudinal analysis of perceived control, coping and psychological distress and also the measurement of birth outcomes. The delivery experience could also be investigated as this is something participants in this and other studies [15] report being concerned about. Other post-partum outcomes such as attachment or infant bonding could also be investigated. Future research should also focuses on recruiting women from low SES and ethnic minority backgrounds, as these women are known to be at greater risk for adverse stress related birth outcomes [6, 8], and for contracting Covid-19 [94, 95].

## Conclusion

The results from this study suggest that pregnant women in Ireland experienced increased levels of psychological distress during the Covid-19 pandemic. The findings also suggest that perceptions of control and avoidant coping are associated with psychological distress in this group and could be used as intervention targets.

## Data Availability

The datasets collected and analysed during the current study are available from the corresponding author on reasonable request.

## Abbreviations

CBT: Cognitive Behaviour Therapy
GAD-7: Generalised Anxiety Disorder-7 scale
EPDS: Edinburgh Postnatal Depression Scale
PSS: Perceived Stress Scale
PDQ: Prenatal Distress Questionnaire
